# The use of a multiple imputation method to investigate the trends in Histologic types of lung cancer in Songkhla province, Thailand, 1989–2013

**DOI:** 10.1186/s12885-016-2441-8

**Published:** 2016-07-04

**Authors:** Hutcha Sriplung, Seesai Yeesoonsang, Edward McNeil, Surichai Bilheem

**Affiliations:** Epidemiology Unit, Faculty of Medicine, Prince of Songkla University, Songkhla, 90110 Thailand; Field Epidemiology Training Program, Bureau of Epidemiology, Department of Disease Control, Ministry of Public Health, Nonthaburi, 11000 Thailand

**Keywords:** Lung cancer, Songkhla, Thailand, Incidence, Squamous cell carcinoma, Adenocarcinoma, Multiple imputation

## Abstract

**Background:**

The incidence of lung cancer in many parts of the country as shown in cancer registry statistics is not decreasing. The incidence of adenocarcinoma (ADCA) in Songkhla is now higher than that of squamous cell carcinoma (SCC) in both sexes. The percentage of the unknown histologic type of lung cancer in Songkhla is around 30 %. The objective of this study is to estimate trends in incidence of the two major histologic types of lung cancer: SCC and ADCA, in Songkhla province of Thailand from 1989 to 2013.

**Methods:**

Age-standardized incidence rates (ASR) were used to compare and described the trends in both major types of cancers. Multinomial logistic regression models were used to impute unknown histological cancer types using a multiple imputation (MI) method to account for the high percentage of unknown histology.

**Results:**

The multinomial predictive model for major types of lung cancer in Songkhla consisted of sex, age, year of diagnosis, and place of residence. After MI, the number of cases with both SCC and ADCA in both sexes increased by one-third of the number of cases with originally known histology. The increasing trends were observed in ADCA in both sexes while SCC in males was stable and in females was decreasing.

**Conclusions:**

A rapid increase in the incidence of ADCA was found while the incidence of SCC in males showed no significant change and it was declining in females. These results warrant an investigation into risk factors other than cigarette smoking. The number of cases has limited use when the age structure of the population under study is changing. Year of diagnosis was one of the predictors in the MI model.

## Background

Primary lung cancer (ICD-10 C33-34) has been the second leading cancer in males and the fourth or fifth in females in Thailand for many decades [1]. In the period of 2007–2009, the estimated age-standardized incidence rate (ASR) was 26.2 and 11.5 per 100,000 Thai male and female populations, respectively [2].

Cancer registries must exclude metastatic cancers to the lungs from being classified as primary lung cancers. It is possible that cancer registries misclassify metastatic lung cancers as primary lung cancers, especially in cancer registries in which the percentage of morphologically verified cases (%MV) is low, and the percentage of cases reported by death certificate only (%DCO) is high [3].

In Thailand, the number of lung cancer cases reported from cancer registries published in the series of ‘Cancer in Thailand’ had low %MV, and many had high % DCO [2]. This meant that there was a high proportion of unknown histologies among lung cancer cases in some registries. Among cases with known histology, the proportion of adenocarcinoma (ADCA) varied from 40 to 70 % of all lung cancer cases, and that of squamous cell carcinoma (SCC) from 20 to 30 % in males, while ADCA varied from 60 to 80 % and SCC from 6 to 20 % in females. According to this figure, the average percentage of ADCA in Thailand is higher than in western countries while the proportion of SCC is much lower. However, a direct comparison is inappropriate when the %MV is low and/or the %DCO is high as occurs in some registries in Thailand. The ratio of the proportion of SCC/ADCA in both sexes was less than 1 in all SEA countries [4].

Songkhla is a province in the southern region of Thailand occupying an area of 7,392 square km on the eastern side of the Malay Peninsula. Lung cancer has been the leading cancer in males since the establishment of the Songkhla cancer registry in 1989 [1, 5, 6]. The incidence of lung cancer in men and women increased from 16.3 and 4.3 in 1989–1991 [7] to 24.4 and 8.1 in 2007–2009 [2], respectively. Increases have also been observed in other regions of Thailand.

Non-small cell carcinoma (NSCC) of the lung is a collective term for carcinoma, not otherwise specified, which includes SCC, ADCA, and other rare primary carcinomas, of the lung. SCC and ADCA are usually reported separately while classification of other types of NSCC tends to be reported inconsistently depending on the cancer registry [8, 9]. When the term NSCC was first introduced, there was a tendency for pathologists to overuse this diagnosis when special molecular studies were not available or completed such as in cytology and tiny biopsy specimens. Recently, a new classification of lung cancer was proposed, and some new terms were introduced [10, 11]. However, SCC and ADCA are still the main types.

A limitation of a cancer registry in estimating an incidence rate ratio is the high rate of missing histological diagnoses. The missing data on specific histological diagnosis usually happens in deep organs such as lung, liver, and brain. It occurs in situations that the patients present in late stage and the aim of treatment by physicians is just for palliation, thus, complete investigation for specific histologic type is not necessary. This situation is still true nowadays in low and middle income countries (LMIC) such as Thailand. So, the missing data on the histology is correlated with the stage and clinical performance of the patients but not directly with the histology of the disease itself. Data are said to be missing at random (MAR) if the probability that a value is missing may depend on observed values in the data but not additionally on the missing value itself [12, 13].

Usually the histological type of cancers in an organ is related to different risk factors, disease course and treatment, thus, the need of the incidence and trends in the cancer of specific histologic type that are closer to the truth than that obtained by the ignorance of the unknown histology is necessary for disease control and medical care planning. MI is a means for representing uncertainty in missing data, by producing a distribution of plausible values for a missing variable in a record, given the values of that record’s non-missing covariates [14]. The process of running a multiple imputation is explained in the Methods section below. The method has been used by cancer registries in Australia to estimate the incidence, mortality, and survival in Aboriginal people [14]. MI produces a distribution of plausible values for a missing variable in a record, given the values of that record’s non-missing covariates. Thus, the MI technique can be used to estimate the plausible proportion of histological type distribution within a cancer registry so that the distribution and age-standardized incidence rates of the histologic type of lung cancer can be calculated with a high confidence of accuracy and are useful for health policy planning. However, to achieve a narrow probability interval of the estimate, the percentage of missing cases must not be too high.

The objective of this study is to estimate gender specific trends in incidence of the two major histologic types of lung cancer: SCC and ADCA, in the Songkhla province from 1989 to 2013 using the multiple imputation method.

## Methods

### Lung cancer cases

We recruited all primary lung cancer cases diagnosed between January 1, 1989 and December 31, 2013. Morphologic diagnoses were grouped into 6 International Code for Diseases – Oncology (ICD-O) categories according to the International Rules for Multiple Primary Cancer [15]: SCC, ADCA, large cell carcinoma (8012/3-8014/3), non-small cell carcinoma, not otherwise specified (NSCC-NOS, 8046/3), other known histology, and unknown histology (8000/3-8011/3). The retrieval of the data was approved by the Ethics Committee of the Faculty of Medicine, Prince of Songkla University.

### Population denominators

Population denominators to calculate incidence rates were estimated from the three population censuses conducted by the National Statistical Office in 1990, 2000, and 2010 [16–18]. Intercensus populations for the years in between were estimated using a log-linear function between two consecutive censuses. The populations beyond 2010 to 2030 were estimated and reported by the Office of the National Economic and Social Development Board [19].

### Statistical analysis

Descriptive statistics for variables in the cancer registry were presented as frequency counts and percentages. Geometric means and standard deviations (sd) were presented for variables having an asymmetric distribution. All analyses were conducted with R [20].

### Multiple imputation method

Multivariate Imputation by Chained Equations (mice) package [21] in R was used to perform the imputation of histological types of lung cancers. We used a polytomous regression imputation method since the histological types are unordered categorical data. The function imputes categorical response variables by the Bayesian polytomous regression model. Since there were cases with true unknown histology, we had to distribute those cases into one of the four categories; SCC, ADCA, large cell carcinoma, other known histology and NSCC. The fact that NSCC is a vague terminology in lung cancer pathology that can fall into one of the 4 histologic types mentioned before, the second step of MI was needed to specify which histologic type in a patient was. So, there were two steps of imputation performed in this study. In the first step, cases with unknown histology were replaced with one of the known histological categories, including NSCC, according to the probability distribution of the groups among those who had known histology obtained by the chained equation method plus a degree of random error. A multinomial (polytomous) logistic regression model was used for generating the distribution according to the predictive ability of basic variables present in the registry database such as year and age at diagnosis, religion, and district of residence. A polytomous regression model is given by:

$$ \log \left({\pi}_j(X)/{\pi}_j(X)\right)={\alpha}_j+\beta {\hbox{'}}_jX, $$

where *α*_*j*_ is a constant and *β'*_*j*_ is a vector of regression coefficients of X explanatory variables, for *j* = 1, 2,…, *J* – 1. [22] The method described by White et al. [23] was applied to avoid bias due to perfect prediction.

In the second step, cases diagnosed with NSCC in the original data, as well as those imputed from the first step, were replaced with SCC, ADCA, large cell carcinoma or other specified histology using a similar approach as the one used in the first step. After the second imputation step, large cell carcinoma and all other specified histological types, including small cell carcinoma and sarcoma, were grouped as ‘other’. Thus, only three categories: SCC, ADCA, and other were obtained.

This two-step multiple imputation method was repeated 200 times to get estimates of the 95 % Bayesian probability interval (PI) or credibility interval obtained from the quantiles of the posterior distribution for the three histologic categories.

### Computation of age-standardized incidence rates

Since comparison of the proportion of SCC and ADCA over a long period can be biased by the change in the age structure of the population, we used the age-standardized incidence rates (ASR) for the two groups to illustrate only the effect of time on the imputed number cases and the trends in incidence rate and to ignore the change in the age structure of the population. The rates were calculated for 24 calendar years from 1989 to 2013. ASRs standardized to the world population modified by Doll [24] in 1966 from that proposed by Segi [25] in 1960 were estimated for each particular year.

### Age period cohort model

Age-period-cohort (APC) regression models were used to investigate the effect of age, calendar year and birth-cohort on the incidence of cervical cancer. We used the classical method which fits a log-linear model with a Poisson distribution to the observed data to estimate age, period and cohort effects in a multiplicative APC model as follows:

$$ log\left[R\left(a,p\right)\right]=f(a)+g(p)+h(c), $$

where the expected log-incidence rates *R(a,p)* is assumed to be equal to a linear combination of effects that adjust for age a, period p and birth-cohort c, where *c = p-a*. To address the non-identifiability problem of the APC models, two-effects models (age-period and age-cohort) were first chosen and the remaining effect (cohort or period) was then identified to the respective model’s residuals using natural splines to reduce random variation [26]. These are referred to as the AP-C and AC-P models. The analysis of APC models was performed with the Epi package [27] for R statistical software version 3.2.2 [28].

## Results

There were 2,734 male and 1,110 female cases with lung cancer diagnosed during 1989–2013. The mean (sd) age at diagnosis was 64.4 (12.4) and 63.8 (14.4) years in males and females, respectively. The distribution of major histological groups of lung cancer cases in both sexes is shown in Table 1. The percentage of SCC was higher in males than females (21.4 vs. 8.4 %) while the percentage of ADCA was higher in females than males (52.5 vs. 33.2 %). The difference in histologic type distribution, sex, district, and period at diagnosis were different between males and females.

**Table 1:** Distribution of basic characteristics of lung cancer cases

	Male		Female		*p*-value*
	Number	Percent	Number	Percent	
Histology	2734	100.0	1110	100.0	<0.001
Squamous cell carcinoma	585	21.4	93	8.4	
Adenocarcinoma	908	33.2	583	52.5	
Non-small cell carcinoma	131	4.8	41	3.7	
Other	219	10.7	71	6.4	
Unknown	791	28.9	322	29.0	
Age (mean, SD)	64.5	12.4	63.8	14.4	<0.605**
Religion					<0.001
Buddhist & others	2328	85.1	1004	90.4	
Muslim	406	14.9	106	9.6	
District					0.047
Muang Songkhla	455	16.6	161	14.5	
Sathing Phra	101	3.7	56	5.1	
Chana	194	7.1	77	6.9	
Na Thawi	135	4.9	41	3.7	
Thepha	116	4.2	42	3.8	
Saba Yoi	64	2.3	21	1.9	
Ranot	150	5.5	71	6.4	
Krasae Sin	30	1.1	14	1.3	
Rattaphum	113	4.1	46	4.1	
Sadao	265	9.7	79	7.1	
Hat Yai	782	28.6	355	32.0	
Na Mom	47	1.7	23	2.1	
Khuan Niang	58	2.1	37	3.3	
Bang Klam	45	1.6	17	1.5	
Singhanaknon	141	5.2	55	4.9	
Khlong Hoi Khong	38	1.4	15	1.3	
Year interval					<0.001
1989–1993	272	10.0	92	8.3	
1994–1998	336	12.3	130	11.7	
1999–2003	434	15.9	178	16.0	
2004–2008	794	29.0	257	23.2	
2009–2013	898	32.8	453	40.8	

* Chi square test

** Wilcoxon rank sum test

Table 2 shows the trends of SCC and ADCA in both sexes. The distribution of SCC and ADCA varied by year of diagnosis. The NSCC appeared by the end of the period during 1994–1998, this histologic diagnosis contributed approximately 5 % of the diagnosis in both sexes. The percentage of the unknown category was around 30 % in both sexes without significant change in the trend according to sex and period while there was a declining trend in other known histologic types throughout all periods.

**Table 2:** Distribution of histologic categories by sex and 5-year periods

Period	SCC	ADCA	NSCC	Other	Unknown
Male					
1989–1993	102 (29.3)	90 (25.9)	0 (0.0)	52 (14.9)	104 (29.9)
1994–1998	97 (29.9)	107 (33.0)	1 (0.3)	44 (13.6)	75 (23.2)
1999–2003	136 (26.5)	172 (33.5)	25 (4.9)	69 (13.4)	112 (21.8)
2004–2008	145 (18.0)	266 (33.0)	44 (5.5)	72 (8.9)	280 (34.7)
2009–2013	105 (14.2)	273 (36.8)	61 (8.2)	73 (9.9)	229 (30.9)
Female					
1989–1993	21 (18.4)	48 (42.1)	0 (0.0)	9 (7.9)	36 (31.6)
1994–1998	16 (11.4)	72 (50.7)	0 (0.0)	17 (12.1)	36 (25.7)
1999–2003	25 (13.6)	92 (50.0)	6 (3.3)	7 (3.8)	54 (29.4)
2004–2008	17 (5.6)	161 (53.3)	17 (5.6)	8 (2.7)	99 (32.8)
2009–2013	14 (3.8)	211 (57.0)	18 (4.9)	16 (4.3)	111 (30.0)

Table 3 shows the predictive multinomial logistic regression model of cases with known histology. Age, sex, and year of diagnosis were the three strongest predictors for ADCA. District of resident was a weak predictor for ADCA. Thus, age, sex, year of diagnosis, and district of residence were included in the multinomial logistic regression model for multiple imputation of lung cancer patients with unknown histology (true unknown histology plus NSCC). Thus, the predictory polytomous model for multiple imputation of unknown histology should include sex and others described above as all of the variables explain the distribution of the known histology cases and should, if the missing at random (MAR) assumption [12] was the case.

**Table 3:** Relative risk ratios for histologic types of demographic variables among cases with known histology

Predictor variable	ADCA	Large cell	Small cell	Others	*P*-value (LR test)
Sex (ref. = Male)	3.92 (3.31–4.65)^a^	1.87 (1.22–2.86)^a^	0.66 (0.39–1.11)	9.11 (9.04–9.18)^a^	<0.001
Age (year)	0.98 (0.98–0.99)^a^	1.00 (0.99–1.02)	1.00 (0.98–1.01)	0.96 (0.93–0.99)^a^	<0.001
Religion (ref. = non-Muslim)	0.98 (0.85–1.13)	0.86 (0.50–1.47)	0.93 (0.58–1.49)	0.99 (0.97–1.01)	0.907
Other district (ref. = Muang)	0.98 (0.96–1.00)	0.62 (0.40–0.95)	0.87 (0.56–1.35)	0.99 (0.90–1.09)	0.084
Year of Diagnosis (ref. <=2000)	2.26 (1.84–2.77)^a^	0.77 (0.55–1.09)^a^	1.72 (1.22–2.43)^a^	1.01 (1.01–1.01)^a^	<0.001

These variables are from the multinomial logistic regression model using SCC as the reference group. Muang district is the old town of Songkhla so it is set as the reference district

^a^Statistically significant with *p*-value < 0.05. *P*-values from likelihood ratio test are given in the last column. LR test = likelihood ratio test

**Fig. 1:**
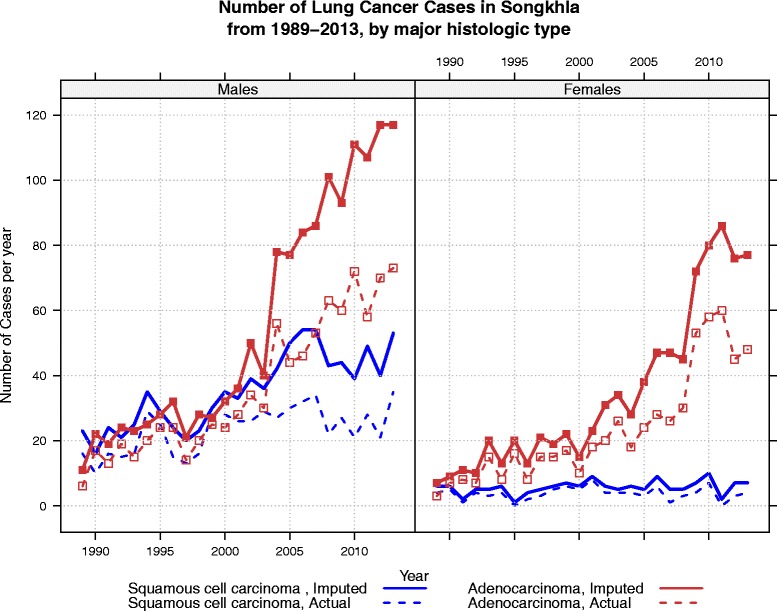
Number of lung cancer cases for the two major histologic types, SCC and ADCA, stratified by sex. The dashed lines represent the number of cases before imputation and the solid lines represent the number of cases after imputation

**Fig. 2:**
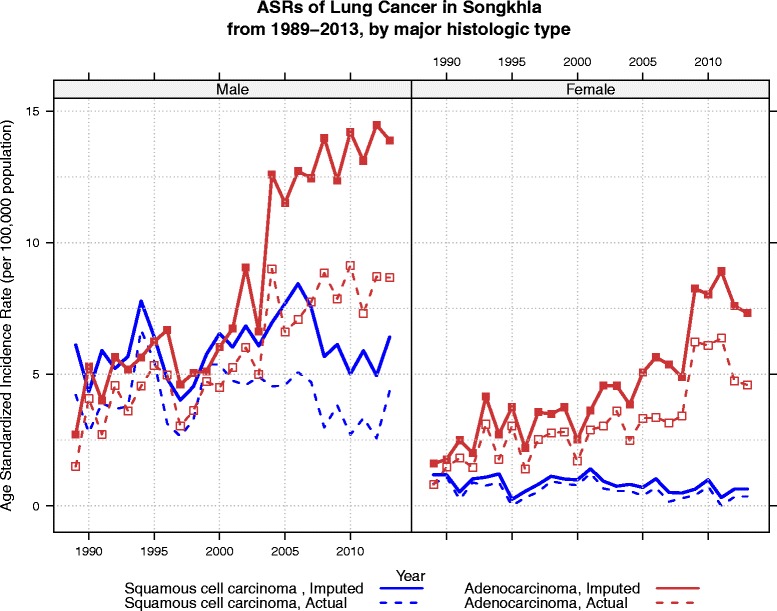
Age standardized incidence rates of lung cancer for the two major histologic types, SCC and ADCA, stratified by sex. The dashed lines represent the rates before imputation and the solid lines represent the rates after imputation

The actual (non-imputed) numbers and percentages of cases with squamous cell carcinoma (SCC), adenocarcinoma (ADCA), and other specified cancer are shown in the first two columns of the Table 4. After running two-step 200 rounds of MI with the polytomous regression model as described in the Method section above for prediction of the histological group in Table 3, the distribution of the estimated numbers and percentages of the three major cancer groups stratified by sex among patients with unknown histology are shown in columns 3 and 4 of the Table 4. Now the distribution of the numbers and percentages in columns 3 and 4 had probability intervals obtained by the 200 rounds of imputation. The table shows the similarity of histologic type distribution of the number of cases with known histology and those with unknown histology, as the results of the MI, with Chi-square p-values of 0.235 and 0.968 for males and females, respectively. This results suggested a little change in the distribution of histologic type from the original distribution after MI. However, it did significantly affect the trends in the number of cases and the incidence rates in terms of absolute values as shown in Figs. 1 and 2. The predicting model in Table 3 showed that the probability of the missing data falling in to SCC and ADCA categories varied with time as time was used as one of the explanatory variables used to run the MI. Figure 1 also shows the increase in the number of known cases of SCC and ADCA over the period 1990 to 2013. To overcome the problem of the change in population age structure that affects the number of cases in different years, the ASR (world) by calendar years from 1990 to 2013 stratified by sex is shown in Fig. 2. With age-period-cohort model, the overall drift (slope) of ADCA in males was 5.9 % per year (95 %CI: 5.1, 6.7 %), that of SCC in males was 0.6 % per year (95 %CI: −0.3, 1.5 %), that of ADCA in females was 6.1 % per year 95 %CI: 5.1, 06, 7.2 %), and SCC in females was −2.1 % (95%CI: −4.4, 0.2 %). Before multiple imputation, the two types of lung cancer in males had almost the same incidence rates and an abrupt change was seen after 2003 where the rates of ADCA rapidly increased while that of SCC slightly declined. After multiple imputation, the incidence of ADCA rose more rapidly than the rate obtained from the cases with known histology. After multiple imputation, the overall trend of SCC was somewhat stable over the whole period. In females, the incidence rates of ADCA behaved in the same way as in males but to a lesser extent, while the rates of SCC declined in cases with known histology.

**Table 4:** Distribution of histological groups by sex

	Known histology			Unknown histology^a^	
	Male	Female		Male	Female
	(*n* = 1812)	(*n* = 747)		(*n* = 922)	(*n* = 363)
Type of cancer			Type of cancer		
Squamous cell carcinoma			Squamous cell carcinoma		
Number	585	93	Number (95 % PI)	296 (263–309)	48 (35–61)
Percent (95 % CI)	32.3 (30.1–34.4)	12.5 (10.1–14.8)	Percent (95 % PI)	32.1 (28.5–35.5)	13.2 (9.6–16.8)
Adenocarcinoma			Adenocarcinoma		
Number	908	583	Number (95 % PI)	482 (454–517)	281 (264–297)
Percent (95 % CI)	50.1 (47.8–52.4)	78.0 (75.1–81.0)	Percent (95 % PI)	52.3 (49.3–56.1)	77.3 (72.7–81.8)
Other specified cancer			Other specified cancer		
Number	219	71	Number (95 % PI)	144 (111–175)	34 (20–51)
Percent (95 % CI)	17.6 (15.0–20.2)	9.5 (6.4–12.5)	Percent (95 % PI)	15.6 (12.0–19.0)	9.5 (5.5–14.1)

Columns are arranged for patients with known histology (real data), and the estimated numbers and percentages by multiple imputation for patients with unknown histology (imputed)

^a^Unknown histology includes NSCC and true unknown histology. The distribution of the number of cases with known and unknown histology cases is not statistically significant with chi-square *p*-values of 0.235 and 0.968 for males and females, respectively. Those with known histology, the true number and the percentage with its 95 % CI (calculated from the standard error) are present. After 200 rounds of multiple imputation, the mean (estimated) number and the percentage of different cancer types among those with unknown histology, and 95 % PI are presented

After imputation, the ratio of SCC/ADCA for the last 5-year period between 2009–2013 using the estimated number of cases was 0.41 and 0.08, for males and females, respectively, which is similar to that obtained by using the estimated ASRs. Figure 1 shows a rapid increase in the number of cases of ADCA after 2004 in both sexes while the rate of increase in SCC in males is much slower than that of ADCA and no change at all in females. When adjusted for age, the increase in ASR of ADCA in both sexes exists but the slope is less than that of the number of cases. There was no obvious change in incidence of SCC among males and a slight decrease of the disease among females.

## Discussion

The term ‘non-small cell carcinoma’ (NSCC) is used when the biopsy tissue is small and pathologists cannot decide whether it is SCC, ADCA, large cell carcinoma, or other specific histologic diagnosis. The term causes difficulty for cancer registries when classifying lung cancer by histologic type. When the percentage of NSCC increases in a cancer registry, the incidence rate and ratio of SCC/ADCA are somewhat unpredictable.

Table 2 shows no change in the percentage of cancers in the true ‘unknown’ category while ‘other’ seems to decrease when NSCC was reported in the last three 5-year periods. Pathologists in Songkhla had been using the term NSCC by the end of the period during 1994–1998, and this histologic diagnosis contributed approximately 5 % of the diagnosis in both sexes. When fine-needle biopsy cytology was introduced in late 1980s, pathologists found it difficult to diagnosis lung cancer based on a smear of individual cells rather than a large piece of tumor tissue. Thus, misclassification is unavoidable when a larger biopsy specimen is not obtained, especially among late stage cancer cases. However, this study shows that it was the true ‘unknown’ category, and not the NSCC, that contributed to the uncertainty of the SCC/ADCA ratio, since the proportion of NSCC was much lower than that of ‘unknown’ category.

Although there was rapid increase in the number of cases of SCC and ADCA both in the actual cases and imputed cases in males and a rapid increase in ADCA in females (Fig. 1), adjustment for population age structure throughout the period, as shown in Fig. 2, suggests a different phenomenon. We clearly show a need to adjust for the population age structure when comparing the change in incidence of cancer over a long period. Age standardization is essential in this case since the predictor model for multiple imputation was proven to be dependent on time. A biased impression on the trends over time is unavoidable when only the number of cases is investigated.

The rather stable ASR of SCC in men and slightly decreasing rate in women and the increasing rates of ADCA in both men and women in Songkhla from 1989 to 2013 are different from trends observed in different subpopulations in the US. The ASR of SCC in the US has been consistently declining while that of ADCA has been stable over the last two decades and have been increasing in the last decade [29–31]. The overall increasing trends in incidence of ADCA and the decreasing trends in SCC have been observed in other western and Asian countries [4, 32–35]. In some Middle Eastern countries, the SCC/ADCA ratio has been decreasing but it still has been high in some areas [36]. As mentioned earlier, the change in diagnosis procedures and classification of lung cancer has contributed partly to the change in the SCC/ADCA ratio [10, 11] and the unsynchronized adoption in various countries around the world may further complicate the story.

A common issue with multiple imputation methods is accuracy in the imputed results. It has been suggested that the data must be missing at random (MAR). One study demonstrated that the linear regression method works well when the percentage of missing values is between 10 and 60 % [37]. However, the study was not dealing with categorical data. A simulation study on multiple imputation using a multinomial logistic regression model showed unbiased results when the values were simulated to be in any of the three missing patterns; missing completely at random (MCAR), missing at random (MAR) and not missing at random (NMAR) gave unbiased results up to the missing data was reaching 80 % [38]. MAR is difficult to be proved and this simulation paper showed that the NMAR data set is acceptable for the imputation of the categorical data type.

In this study, there are no obvious reasons for bias among cases with missing histology. The chance of death among all histologic types is theoretically non-differential. Cases diagnosed by death certificate only (DCO) are not likely to incur bias as the SCC, ADCA, and other histologies have an equally poor prognosis. Clinicians may decide to perform cytology and/or biopsy based on patients’ performance status and compliance, however, there is no direct association between performance status and histologic type of lung cancer. The preference of pathologists to diagnose a particular histologic type over another is an important source of bias. The prevailing use of immunohistological staining in pathological investigation may raise the possibility that ADCA might have been diagnosed more often in recent years than in the past. However, a study in Japan showed no differential misclassification in retrospective review of slides in the past against recently diagnosed cases [39].

## Conclusions

With the use of multiple imputation methods, we observed a rapid increase in incidence of ADCA, which outnumbered that of SCC in both sexes over the period of 1989–2013 in Songkhla. The incidence of SCC in males showed no significant change throughout the period while it was declining in females. We also showed that it is necessary to use age standardized rates when studying trends over a long period, especially when the predictive model for MI is dependent on the period under study.

## Abbreviations

95 % CI, 95 % confidence interval; 95 % PI, 95 % probability interval; ADCA, adenocarcinoma; ASR, age-standardized incidence rate; DCO, death certificate diagnosis only; ICD-O, International Codes for Diseases - Oncology; MAR, missing at random; MI, multiple imputations; MV, morphologically verified; NOS, not otherwise specified; NSCC, non-small cell carcinomas; SCC, squamous cell carcinoma; Sd, standard deviation
